# Draft Genome Sequence of Haloalkaliphilic *Exiguobacterium* sp. AB2 from Manleluag Ophiolitic Spring, Philippines

**DOI:** 10.1128/genomeA.00840-14

**Published:** 2014-08-14

**Authors:** Gamaliel Lysander B. Cabria, Vina B. Argayosa, Jose Enrico H. Lazaro, Anacleto M. Argayosa, Carlo A. Arcilla

**Affiliations:** a National Institute of Molecular Biology and Biotechnology, University of the Philippines Diliman, Quezon City, Philippines; b Natural Science Research Institute, University of the Philippines Diliman, Quezon City, Philippines; c Institute of Biology, University of the Philippines Diliman, Quezon City, Philippines; d National Institute of Geological Sciences, University of the Philippines Diliman, Quezon City, Philippines

## Abstract

*Exiguobacterium* sp. AB2 is a haloalkaliphilic bacterium isolated from a hyperalkaline spring in Manleluag, Pangasinan, Philippines. Sequencing of bacterial DNA assembled a 2.85 MB draft genome. Analysis suggests the presence of genes for tolerance to stresses such as elevated pH and salt concentrations and toxic metals.

## GENOME ANNOUNCEMENT

*Exiguobacterium* spp. have been found in many extreme environments such as Antarctic ice (1), Himalayan glaciers (2), soda lakes (3), hyperthermophilic hot springs (4), and deep-sea hydrothermal vents (4, 5). They have been reported to persist in paper mills and in rhizospheres (6, 7). They have also been identified as opportunistic nosocomial pathogens (8).

The isolate *Exiguobacterium* sp. AB2 was isolated from a serpentinized ultramafic hyperalkaline spring in the Manleluag Protected National Park, Mangatarem, Pangasinan, Philippines (15°42′16″ N and 120°16′52″ E). The spring is situated at the eastern periphery of the Zambales Range and within the Zambales Ophiolite Complex. The spring has a characteristic “rotten egg” odor and blue flame ignition from the gas bubble emissions, indicating high methane and sulfur content, and is associated with travertine terrace formations. Water pH and temperature (pH 10 to 11.26 at 33 to 36°C) vary with large daily fluctuations in water level (9).

*Exiguobacterium* sp. AB2 is a non-spore forming, pleomorphic, Gram-positive, extracellular protease-producing bacterium that can grow from 12°C to 45°C and can tolerate alkaline (7<pH<11) and haline (0 to 15% wt/vol NaCl and 0 to 10% wt/vol MgCl_2_) conditions. Based on 16S rRNA gene phylogeny, the isolate clusters with other hot spring and marine isolates (Group II) within the *Bacillales* family XII *incertae sedis, Firmicutes* (4).

Whole-genome shotgun sequencing was done using the Roche GS Junior Platform. Sequencing generated 164,991 single-end reads in a total of 71,162,194 bases. Reads were assembled using the Newbler 2.5p1 (10) in the GS Junior De Novo Assembler resulting in 2,853,500 bases assembled in 114 contigs, and with a G+C content of 52.92%. With an *N*_50_ contig size of 50,442 bp and mean coverage of 23, the estimated complete genome size of *Exiguobacterium sp.* AB2 was 3.1 MB. Therefore, sequencing covered an estimated 92.05% of the expected genome.

Gene prediction and genome annotation were done using glimmer3.0 in the RAST-NMPDR server (11, 12) and GeneMarkS+ through the NCBI Prokaryotic Genome Annotation Pipeline (PGAP 2.5) (13). RNA features were predicted using PGAP and also RNAmmer and tRNAscan-SE (14, 15). The number of non-coding RNAs predicted is 74 sequence variants comprising 8 rRNA and 66 tRNA sequences (PGAP: 9 rRNA and 64 tRNA).

The number of putative genes was estimated at 2,963 genes with 2,836 coding sequences and distributed in 398 predicted SEED subsystem features in RAST. A HMMER domain search of the genome revealed several enzymes of possible commercial interest, including an extracellular alkaline protease, cellulase, and chitinase among others. Stress tolerance genes found by RAST annotation of the genome include operons for arsenic and mercuric metabolism, cobalt-zinc-cadmium resistance genes, and various antibiotic resistance genes. Transport genes for magnesium, sodium, cation/proton antiporters, and choline-betaine uptake and synthesis were also predicted, which might explain the salt tolerance. Along with anti-porters, carbonic anhydrase was also predicted, which might play a role in pH regulation and biomineralization of carbon dioxide (16).

This report is the ninth genome announcement for the genus *Exiguobacterium*. The sequence will add to our knowledge of the bacterium’s role in the environment and of its possible applications in biotechnology.

### Nucleotide sequence accession numbers.

This whole-genome shotgun project was deposited at DDBJ/EMBL/GenBank under the accession no. JNAA00000000. The version described in this paper is version JNAA01000000.
